# Delayed Presentation of Basal Cell Carcinoma: A Case Report

**DOI:** 10.7759/cureus.10695

**Published:** 2020-09-28

**Authors:** Andre A Abadin, Scott Fosko, Michael Boniface, Richard J Presutti

**Affiliations:** 1 Family Medicine, Mayo Clinic, Jacksonville, USA; 2 Dermatology, University of Florida, Gainesville, USA; 3 Emergency Medicine, Mayo Clinc, Jacksonville, USA; 4 Medicine, Mayo Clinic, Jacksonville, USA

**Keywords:** basal cell carcinoma, skin cancer, bcc, malignant, indolent skin lesion

## Abstract

Basal cell carcinoma (BCC) is the most common skin cancer in the United States. Although BCC has a low metastatic potential, it can be locally invasive and destructive, especially when there is a delay in diagnosis or treatment. This can affect not only the surrounding skin, but deeper tissues including muscle, cartilage, and even bone. Primary care physicians often serve as the first line of defense in the recognition, diagnosis, and even treatment of skin lesions suspicious for BCC. Most low-risk BCC can be treated in the primary care office with electro-desiccation and curettage or surgical excision. We present a case of locally invasive BCC with significant soft tissue destruction of the neck, which was incidentally identified during an emergency department presentation for a myocardial infarction. It is the responsibility of primary care physicians to recognize the appearance of skin lesions suspicious for BCC and initiate or arrange for subsequent definitive diagnosis and treatment. Our intent in presenting this case is to illustrate a missed opportunity for earlier recognition and treatment because of lack of access to primary care, as well as to demonstrate the destructive nature of BCC when neglected over time. Comprehensive approaches to diagnosis and treatment are described elsewhere.

## Introduction

Skin cancer is the most frequently diagnosed malignancy in the United States with annual incidence exceeding 5 million cases [1]. Among these, basal cell carcinoma (BCC) is the most frequent histologic diagnosis. Previous epidemiologic studies have identified predictable risk factors for the development of BCC including fair skin color, excessive sun exposure, and use of tanning beds [2]. Although BCC has low metastatic potential relative to other skin malignancies such as melanoma, reduction of risk factors and early detection remain the foundation of prevention and disease management. Despite the low metastatic potential, BCC may still result in significant morbidity due to local invasion and soft tissue destruction, in some cases requiring wide surgical excisions and complex reconstruction. In fact, the primary factor responsible for increased morbidity in BCC is a delay in diagnosis or treatment [3,4]. Herein, we report a case of a 70-year-old male with locally invasive BCC affecting the sternocleidomastoid and possibly trapezius muscles who had not sought medical evaluation for over 15 years and whose lesion was only identified incidentally during an emergency department presentation for a myocardial infarction.

## Case presentation

**Figure 1:**
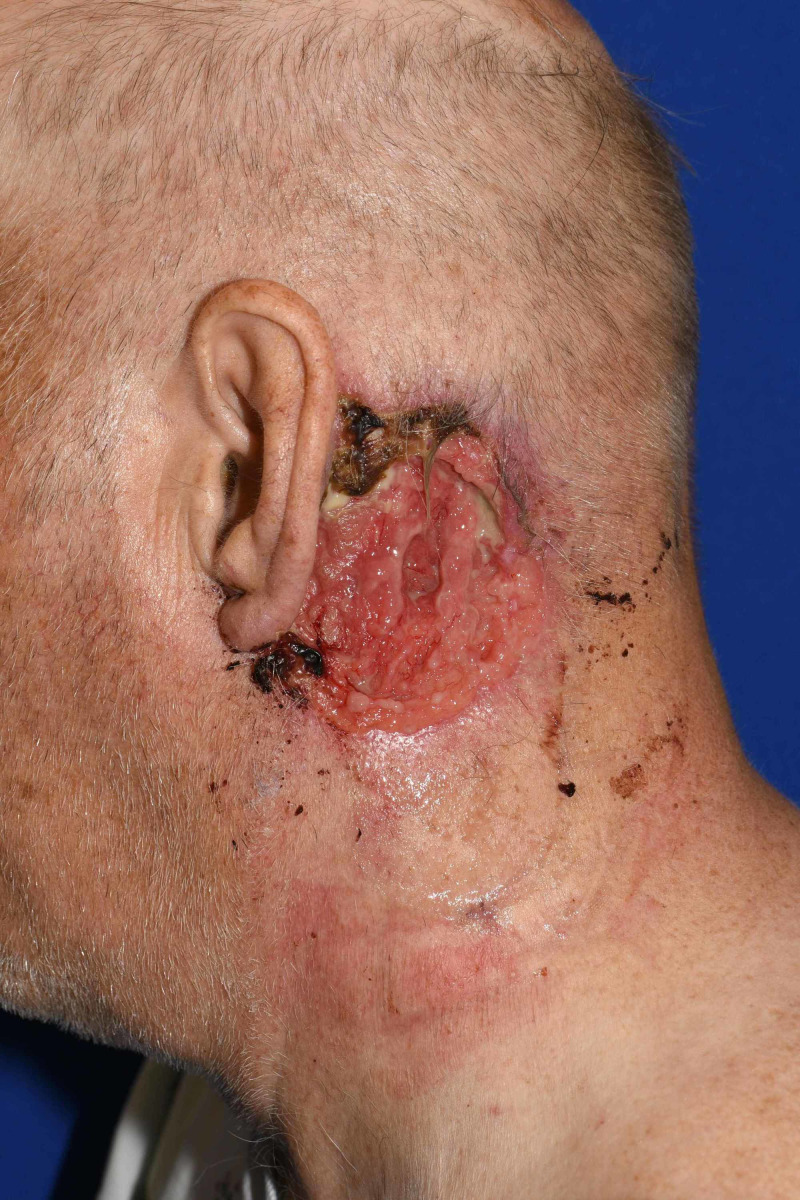
7.5 x 6.0 cm ulcerated plaque with purulent drainage on the left postauricular area

**Figure 2:**
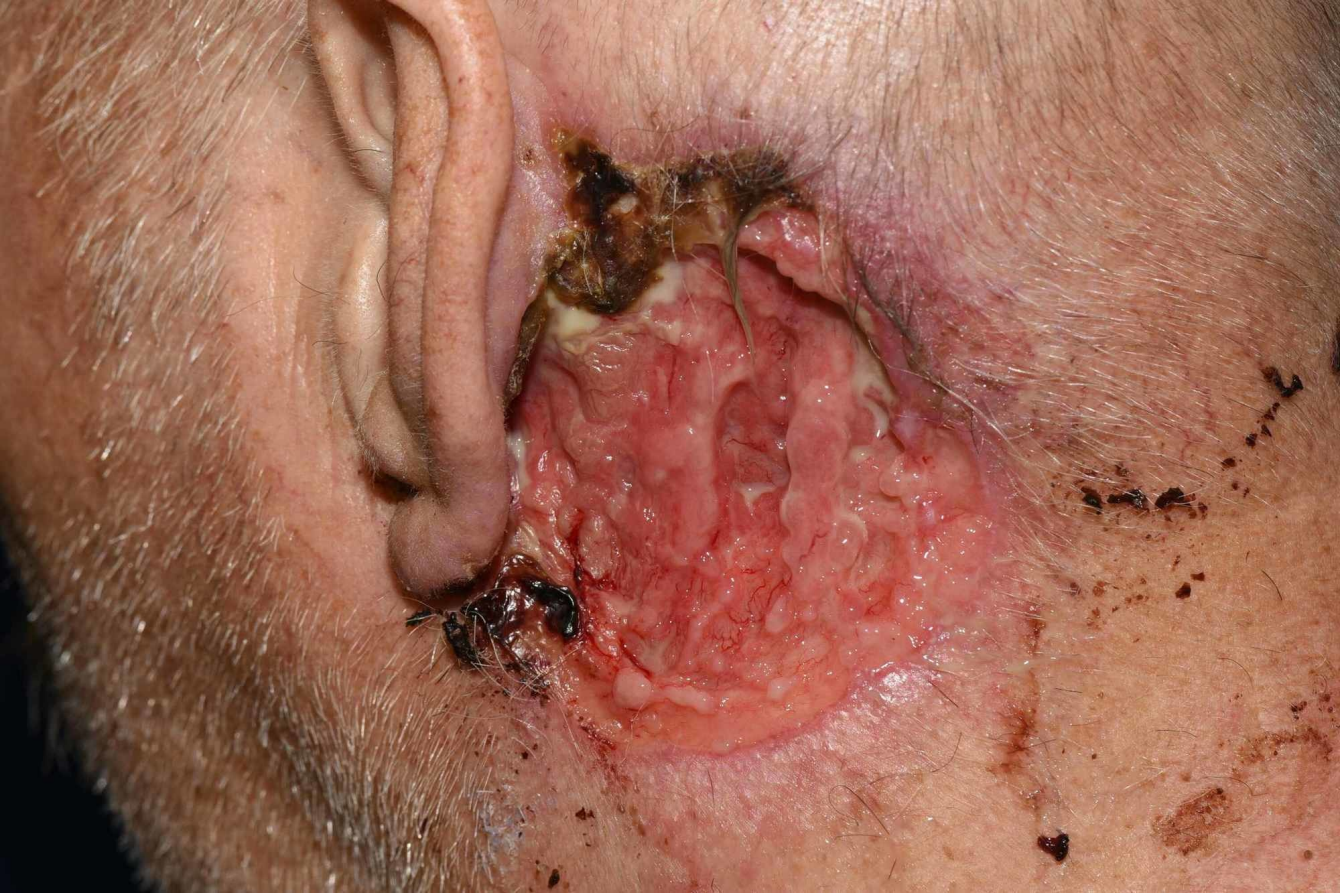
A closer view of the 7.5 x 6.0 cm ulcerated plaque with purulent drainage on the left postauricular area

The patient is a 70-year-old Caucasian male with no known past medical history who presented to the emergency department (ED) by rescue with syncope and appeared acutely ill. He had not seen a physician for over 15 years. He has a smoking history of 50 pack year. During initial assessment in the ED, he was found to have a large, bleeding, ulcerative wound posterior to his left ear. However, diagnostic evaluation for his presenting complaint was notable for ischemic ST depressions in the inferior and anterolateral electrocardiogram leads and up trending serum troponins, resulting in the diagnosis of acute coronary syndrome. He was admitted to the hospital and treated for a non-ST elevation myocardial infarction with subsequent percutaneous coronary intervention and stent placement. When his condition stabilized from a cardiac perspective, additional history revealed that a “red rash” initially developed adjacent to his left ear about 15 years ago. The rash grew slowly and ultimately became ulcerated with purulent discharge. His wound care at home consisted of washing the area with soap and water and letting it air dry. Physical exam revealed 7.5 x 6.0 cm ulcerated plaque with rolled borders, peripheral hemorrhagic crust, and purulent drainage on the left postauricular area and upper neck (Figures 1, 2).

Also, the examination of his left upper eyelid revealed a waxy, pink plaque with telangiectasia. A punch biopsy of both lesions confirmed basal cell carcinoma. CT of the neck with contrast showed invasion of the ulcerated lesion into the distal left sternocleidomastoid muscle and its insertion, and loss of the fat plane between the ulcerative lesion and upper left trapezius muscle. Surgical resection was recommended with the possibility of adjuvant hedgehog inhibitor therapy with vismodegib treatment conditional on lymph node involvement. His surgery date was delayed due to repeated hospitalizations for complications of ischemic cardiomyopathy and ultimately, he expired prior to intervention five months following index presentation.

## Discussion

BCC has a 20% lifetime risk of development [2], and is known to be slow growing with rare metastasis [4,5]. For this reason, the risk of basal cell carcinoma to cause functional loss and decrease quality of life is often overlooked. As this case demonstrates, delay in care resulted in advanced local invasion with need for complex surgical intervention. The patient had detected an abnormal skin lesion years prior to his local invasive presentation, but failed to establish a primary physician to inspect the lesion to determine if further workup was necessary. Although this patient’s tumor was believed to be still amenable to surgical resection, there are times where BCC can cause local destruction to a point where resection is no longer a viable option [2].

Due to its low malignant potential and slow growth, it has been suggested to change BCC to an “indolent lesion of epithelial origin” [6]. This proposed name change to replace carcinoma with indolent lesion would minimize the potential damaging consequences of BCC. Moreover, the removal of the word carcinoma will have potentially dangerous effects on how health-care professionals and the public perceive the dangers of BCC [7]. Physicians and patients might misinterpret this shift in verbiage and delay their presentation or treatment. Late presentation to a physician occurs for several reasons including a skin lesion located elsewhere than the head or neck, a skin lesion not associated with bleeding or itching, those patients younger than 65 years old, no family history of skin cancer, and denial of their skin lesion [8]. This patient exemplified a few of the reasons: an initial skin lesion that did not bleed or itch, an absent family and personal history of BCC, and a denial of his skin lesion.

An initial presentation of skin cancer in the emergency department, like this patient, is not ideal, but prevalent. For many patients, the emergency department is the initial point of entry into a complex health care system. In a retrospective cohort of patients undergoing CT evaluation of the abdomen for any reason, incidental occult malignancies were detected with 2% incidence [9]. Beyond incidental findings on diagnostic imaging, diagnosis of cancers is common in the emergency department and is associated with worse outcomes than when detected through screening [10]. Education is necessary for patients to understand the importance of how early detection can reduce the burden of delayed treatment [11].

Primary care physicians are well positioned in the healthcare system to educate patients on BCC, as well as other skin cancers. They are often the first physicians to encounter and diagnose skin lesions [12]. Although the U.S. Preventive Services Task Force has found no evidence for or against recommendation on performing full body examinations to screen for skin cancer, they do suggest vigilance to remain alert for any suspicious lesions through the patient’s history and physical examination [13]. When identified, definitive diagnosis is obtained through skin biopsy. Dermoscopy should be considered by appropriately trained providers as it is noninvasive, improves pre-biopsy accuracy, and performs with increasing frequency by primary care physicians [14]. Physicians should familiarize themselves with clinical subtypes and characteristic features of BCC, which can help group BCCs into low-risk or high-risk (Table 1).

**Table 1:** Basal cell carcinomas (BCCs) low-risk group vs high-risk group. Adapted with permission from the NCCN Clinical Practice Guidelines in Oncology (NCCN Guidelines®) for Basal Cell Skin Cancer V.1.2020. © 2020 National Comprehensive Cancer Network, Inc. All rights reserved. The NCCN Guidelines® and illustrations herein may not be reproduced in any form for any purpose without the express written permission of NCCN. To view the most recent and complete version of the NCCN Guidelines, go online to NCCN.org. The NCCN Guidelines are a work in progress that may be refined as often as new significant data becomes available [14-16].

Clinical Presentation	Low Risk	High Risk
Size on trunk and extremities	Less than 10 mm	10 mm or greater
Size on cheek, forehead, neck, and pretibia	Less than 20 mm	20 mm or greater
Borders	Well defined	Poorly defined
Immunosuppressed	Negative	Positive
Primary or Reoccurrence	Primary	Recurrent
Perineural Involvement	Negative	Positive
Histology Subtypes	Nodular, superficial, and nonaggressive growth patterns: keratotic, infundibulocystic, and fibroepithelioma of Pinkus	Morpheaform, basosquamous, sclerosing, mixed infiltrative, or micronodular

Understanding the information provided in the pathology report is also critical to properly categorize BCCs [5,15,16]. Low-risk BCC can be treated by a primary care physician with proper training and knowledge most often with surgical excision or in select cases with electro-desiccation and curettage [14,15]. BCC with high-risk features should be referred for evaluation by a dermatologist for consideration of Mohs surgery or adjuvant therapies (Table 2).

**Table 2:** Treatment options for basal cell carcinoma (BCC). Adapted with permission from the NCCN Clinical Practice Guidelines in Oncology (NCCN Guidelines®) for Basal Cell Skin Cancer V.1.2020. © 2020 National Comprehensive Cancer Network, Inc. All rights reserved. The NCCN Guidelines® and illustrations herein may not be reproduced in any form for any purpose without the express written permission of NCCN. To view the most recent and complete version of the NCCN Guidelines, go online to NCCN.org. The NCCN Guidelines are a work in progress that may be refined as often as new significant data becomes available [5,14-19].

Therapy	Considerations
Standard surgical excision with postoperative margin evaluation (SSEPME)	Gold standard for low-risk BCC
Mohs micrographic surgery	Lowest recurrence rates for high-risk BCC
Electrodesiccation and curettage	Fast, cost-effective, convenient. Can be performed by appropriate trained primary care physicians
Topical therapies	Includes 5-fluorouracil and imiquimod as common agents
Intralesional therapies	May be more efficacious than topical administration
Cryosurgery	Poor cosmetic outcomes compared with SSEPME, lacking strength of evidence. Use of thermocouples recommended.
Photodynamic therapy	For low risk BCC. Less efficacious than SSEPME, but better cosmetic outcomes
Laser Therapy	May be monotherapy or adjuvant
Radiotherapy	Especially helpful for non-surgical candidates. Preferentially target malignancy while preserving viable tissue.
Systemic chemotherapy and immunotherapy	For locally advanced disease and metastatic disease. Hedgehog pathway inhibitor Vismodegib and Sonidegib FDA approved, PD-1 inhibitors Nivolumab and Pembrolizumab under investigation.

It must be emphasized that basal cell carcinomas can have a range of clinical and pathologic presentations, and understanding them is critical to guiding the most appropriate and effective treatment [14-16]. A dermatologist is a critical and valuable resource to assist with these decisions. Patients with suspicious or unusual skin lesions that are concerning for high-risk more aggressive cutaneous malignancies, such as malignant melanoma, should also be referred to a dermatologist.

## Conclusions

This case report highlights the capabilities of BCC to cause local destruction when detection and treatment are delayed. Barriers to early detection may be attributable to patient factors (denial), health system factors (inadequate access to primary care), and provider factors (suboptimal training or experience). There is risk of exacerbating these factors for delay if BCC is renamed to indolent lesion of epithelial origin. It is imperative for primary care physicians to be judicious in their management of skin lesions as they are often the first to encounter skin cancer in its early stage. Timely diagnosis will reduce the physical and psychological burdens of delayed treatment.
